# Skin metastases from prostate cancer successfully treated with radiation therapy

**DOI:** 10.1259/bjrcr.20200142

**Published:** 2023-06-13

**Authors:** Francesco Marampon, Martina Parisi, Piero Rodolfo Cicco, Maria Serpone, Miriam Tomaciello, Daniela Musio, Francesca De Felice, Vincenzo Tombolini

**Affiliations:** 1 Department of Radiological, Oncological and Pathological Sciences, "Sapienza" University of Rome, Rome, Italy

## Abstract

Skin metastases from prostate cancer (PCa) are rare, cause considerable discomfort, and usually indicate advanced disease and a poor prognosis. To date, literature accounts for no more than 88 cases of skin metastasis from PCa, and radiation therapy (RT) is not considered a standard treatment option. Here, we have described a rare case of skin localization of castration-resistant metastatic PCa, which occurred in a 75-year-old male previously treated with RT for PCa, 11 years earlier. The skin lesions, which progressively appeared in different areas of the chest wall, were successfully treated with electron beam RT (900 cGy, for 3 consecutive days). Five months after irradiating skin metastases, the patient showed general fair conditions and no longer developed other skin lesions in the areas already treated or elsewhere. This report describes a scarce case of cutaneous metastases from PCa, underlying the crucial role of RT as a definitive palliative treatment that should be used to limit systemic chemotherapy-related toxicity.

## Introduction

Skin metastases occur in less than 1% of cancer patients^1^ and very rarely in patients with prostate cancer (PCa),^2^ usually indicating advanced disease. Radiotherapy (RT) improves patients' quality of life by relieving symptoms associated with skin metastases such as pain, bleeding, and ulceration.^1^ However, treatment schedules usually used (30–66 Gy in 10–33 fractions)^3^ require many daily sessions, with consequent discomfort for patients who are often already clinically compromised.

To date, of PCa skin metastases described in the literature^4–11^ only.^5^ Herein, we report on a rare case of a 75-year-old male with a metastatic castration resistant PCa successfully treated by extreme-hypofractionated electron RT, with a clinically durable response.

## Clinical presentation

**Figure 1.**
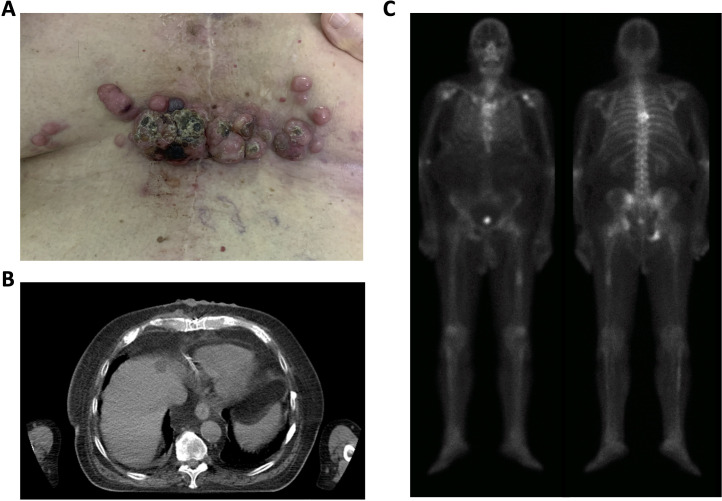
(**A**) Cutaneous lesions at diagnosis. (**B**) CT of skin nodules (**C**) Bone scan.

In September 2019, a 75-year-old male with hormone-refractory metastatic PCa, diagnosed 11 years earlier, in treatment with enzalutamide and already irradiated on painful bones metastases, was referred to our department for palliative therapy of cutaneous metastases located in the sternal region (Figure 1A). The lesions, mostly covered with an easily removable rind, malodorously and easily bleeding, were the cause of great discomfort, pain, and insomnia. A total body CT (Figure 1B) and a bone scan (Figure 1C) respectively showed only skin and bone lesions. The skin lesions were not histologically characterized as the patient refused the biopsy.

## Case management and treatment

**Figure 2.**
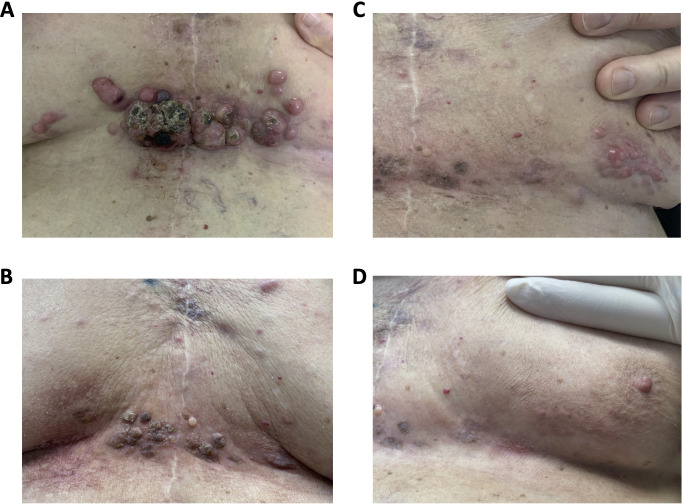
(**A**) Cutaneous lesions at diagnosis. (**B**) Cutaneous lesions in Figure 2A and weeks after irradiation. (**C**) New cutaneous lesions. (**D**) New and old cutaneous lesions, 5 months after treatment.

Skin metastases were treated with 12 Mev electron with 1 cm bolus (100% of the dose at 1 cm from the skin surface) to a total dose of 27 Gy in 3 fractions of 9 Gy, delivered Monday, Wednesday, and Friday of the same week. A 1.5 cm radiation field margin beyond the clinically apparent tumor was used. Compared to before treatment (Figure 2A), lesions had largely regressed two weeks after irradiation (Figure 2B) as well as any kind of symptomatology or disorder previously reported. By January 2020, the treated lesions had completely disappeared although new ones appeared (Figure 2C), which were then successfully treated with the same therapeutic scheme (Figure 2D).

## Outcome and follow-up

As of June 2020, 10 months after the first and five after the second irradiation, the patient was still alive and had no other skin lesions in the treatment field or other locations. The patient died in November 2020 from problems related to COVID-19 infection.

## Discussion

The management of cutaneous metastases requires systemic and/or localized approaches, depending on the primary tumor type, the site of metastasis, the patient’s condition and other ongoing therapies.^2^ RT is widely used in the treatment of skin metastases with a dose range of 30–66 Gy in 10–33 fractions, schedules which not only force patients to prolonged daily treatments but which should be avoided/limited in the COVID-19 era.^3^ Therefore, extreme hypofractionated schemes, very high doses in very few fractions, capable of guaranteeing the same efficacy and better patient compliance, reducing the number of treatments, should be preferred.

The α/β ratio is the radiobiologic parameter explaining the behavior of normal and cancer tissues with respect to radiation schedules.^12^ PCa has been shown to have a very low α/β ratio and therefore is highly responsive to fraction size.^13^ Thus, single larger doses should ensure greater control of the disease and consequently better symptoms efficacy. Interestingly, the only case reported in the literature of a patient with PCa skin metastases was effectively treated with an extreme hypofractionated scheme, 18 Gy in 3 fractions.^5^ Considering an α/β ratio of 1.5 Gy for PCa,^14^ the treatment schedule used by Mak et al ensured a biological effective dose (BED), a measure of the true biological dose delivered by a particular combination of dose per fraction, total dose and specific α/β ratio, of 90 Gy. Since the BED on the tumor should ideally be equal to or greater than 100 Gy, we have decided to enhance this treatment scheme by using 27 Gy in 3 fractions, obtaining a BED equal to 189 Gy. On the other hand, the high α/β ratio of the skin and therefore its lower sensitivity to dose variations resulted in a lower impact in terms of toxicity.

In conclusion, given the high efficacy found and the low toxicity, our experience suggests that the use of an extreme hypofractionated regimen may represent a valid alternative in the treatment of cutaneous PCa metastases.

## Learning points

1. Skin metastases are uncommon, appear many years after the first diagnosis, can be the first manifestation, results in a great deterioration of quality of life and generally herald poor outcome. Treatment provides surgery, chemotherapy and/or radiotherapy.
2. Cutaneous metastases of PCa are rare, usually occur as suprapubic nodules and there is no univocal consent on the treatment approach.
3. The experience herein reported suggests that radiotherapy can provide a durable clinical response with relief from bleeding and pain.
